# Arrival of Oropouche Virus in a Nonendemic Area in Northeastern Brazil, 2024

**DOI:** 10.1002/jmv.70780

**Published:** 2025-12-29

**Authors:** Jean P. M. Nascimento, Thiago P. G. Araújo, Mykaella A. Araújo, Mateus M. G. Arruda, Vitória P. Simplicio, Emelly B. Calheiros, Aline C. Pereira e Silva, Laura M. N. Silva, Marcus R. Santos, Magliones C. Lima, Hazerral O. Santos, Ênio J. Bassi, Alessandra A. Borges, Anderson B. Leite, Abelardo Silva‐Júnior

**Affiliations:** ^1^ Laboratório Central de Saúde Pública de Alagoas Maceió Alagoas Brazil; ^2^ Universidade Federal de Alagoas, Instituto de Ciências Biológicas e da Saúde Maceió Alagoas Brazil; ^3^ Universidade Federal do Vale do São Francisco Paulo Afonso Bahia Brazil; ^4^ Universidade de São Paulo, Faculdade de Medicina Veterinária e Zootecnia Butantã São Paulo Brazil; ^5^ Universidade Federal de Viçosa Viçosa Minas Gerais Brazil

**Keywords:** arboviral disease surveillance, genomic sequencing, glycoprotein Gc mutations, oropouche virus (OROV)

## Abstract

*Orthobunyavirus oropoucheense* (OROV) causes Oropouche fever, which exhibits symptoms similar to those of other arboviral diseases. Although it has historically been restricted to the Amazon region, the virus has recently spread to other areas of Brazil. Alagoas state, with low socioeconomic conditions and limited health coverage, has seen an increase in febrile cases without confirmed molecular diagnoses of circulating arboviruses. By September 6, 2024, 1316 samples negative for Dengue, Zika, and Chikungunya were tested for OROV and Mayaro virus using RT‐qPCR, yielding 115 (8.74%) positive results for OROV. Among these, 14 (22.22%) viral isolates were obtained in Vero cells and confirmed by RT‐qPCR and immunofluorescence assay (IFA). The study generated 37 new near‐complete genomic sequences corresponding to the newly characterized OROV lineage and examined selection pressures on the M gene, identifying sites under purifying selection. We identified amino acid variations in the Gc glycoprotein structure at positions 507, 552, 738, and 795, which may influence host‐cell interactions. This work is the first to report OROV in Alagoas, emphasizing the need for improved monitoring and control measures to mitigate public health impacts.

## 1 Introduction

*Orthobunyavirus oropoucheense* (OROV) belongs to the *Orthobunyavirus* genus. It was first reported in 1955 in Trinidad and Tobago and has since caused outbreaks, primarily in Peru, Panama, and Brazil [1]. In Brazil, OROV was first isolated in 1960 from sloths and *Aedes serratus* near the construction site of the Belém‐Brasília highway [2]. The Amazon region of northern Brazil remains endemic for OROV, with 261 reported cases between 2015 and 2022 [3]. However, in 2023, the Brazilian Ministry of Health reported a significant increase in OROV cases across various regions, totaling 7497 confirmed cases by early September 2024 [4].

OROV is an enveloped virus in the *Peribunyaviridae* family, antigenically related to the Simbu serogroup. It possesses a segmented, negative‐sense single‐stranded RNA (ssRNA) genome consisting of three segments: small (S), medium (M), and large (L) [5, 6]. Among these segments, the M segment encodes the structural glycoproteins Gn and Gc, which are recognized as significant antigenic determinants and have been implicated in mediating host‐cell entry. Additionally, it encodes the non‐structural protein NSm, which plays a crucial role in viral assembly and budding [7]. Successive reassortment events in South America may be related to the rise of the OROV clade (2005–2023), which is spreading in Brazil, characterized by the M segment from the OROVbr clade (2009–2018) and the L and S segments from the OROV Pe/CO/EC clade [8].

In segmented viruses, reassortment is crucial for generating new strains, driving viral evolution, and facilitating adaptation to new hosts. Additionally, these events enable OROV to exploit new ecological niches and adapt to novel vectors [9], facilitating its dissemination to non‐endemic regions. Recent studies have shown that the BR‐2015‐2024 lineage exhibits increased replication in vitro and in animal models, along with partial escape from neutralizing antibodies, suggesting that reassortment events may enhance viral fitness and transmissibility [10, 11]. In urban and peri‐urban regions, OROV transmission is primarily associated with the biting midge *Culicoides paraensis* [12]. The recent outbreaks outside the Amazon may be driven by human factors such as rapid urbanization, inadequate sanitation, and stagnant water accumulation [13]. Evidence suggests that changes in land use, deforestation, and agricultural expansion increase interactions between humans and vectors, which could explain the expansion of OROV beyond its historically endemic areas [14].

OROV causes Oropouche fever, an arboviral disease characterized by high fever, headache, arthralgia, myalgia, skin rashes, malaise, nausea, and vomiting. Rare complications can include meningitis and encephalitis [1]. Recently, there has been increasing evidence of an association between congenital OROV infections and fetal loss, stillbirths, and other complications [15]. During the acute phase of the disease, viral genetic material can be detected in patient samples using molecular techniques such as quantitative reverse transcription polymerase chain reaction (RT‐qPCR) or viral isolation in cell culture [1].

Recent geographic expansion changes have increased the dissemination of OROV beyond the Amazon region [16]. Alagoas, including its coastal geography and adjacent areas, is particularly vulnerable to unexpected outbreaks due to low socioeconomic indicators and limited regional health coverage, despite not being traditionally affected. Considering Brazil's recent Oropouche virus (OROV) outbreak [4] and its potential to expand to new regions, a comprehensive investigation is essential to assess its presence in additional areas. Furthermore, like other arboviruses, Oropouche fever is a neglected disease, highlighting the need for increased attention to its public health implications in Alagoas and across Brazil. Given the increasing number of febrile cases and the lack of confirmed molecular diagnoses for arboviruses, this study aimed to assess the circulation of OROV in Alagoas during 2024. This research is significant because it addresses a critical gap in the surveillance of neglected arboviruses, providing valuable insights to inform public health strategies and control measures.

## 2 Methods

### 2.1 Sample Selection and Molecular Diagnosis

Serum samples from reverse transcriptase quantitative polymerase chain reaction (RT‐qPCR)‐ negative patients for Zika, Dengue, and Chikungunya were tested for Oropouche (OROV) and Mayaro (MAYV) viruses. Viral RNA was extracted using the Extracta® Kit – DNA and RNA of pathogens – MPTA MDx (Loccus) on the Extracta® 96 DNA and RNA extractor and purifier (LOCCUS), following the manufacturer's instructions. The extracted RNA was subjected to RT‐qPCR.

For the molecular detection of OROV and MAYV viruses, the IBMP Mix Fit I ‐ Mastermix OneStep kit (Instituto de Biologia Molecular do Paraná ‐ IBMP) was used, along with previously designed oligonucleotides [17]. All runs were performed on Applied Biosystems™ 7500 real‐time PCR System, with detection based on cycle threshold (Ct) values < 40.

Our study analyzed samples from patients who visited public health units in the state of Alagoas with symptoms suggestive of arbovirus infection. All suspected cases of acute arboviral infections in Alagoas are referred to the state's central laboratory, LACEN‐AL (Alagoas, Brazil). All study procedures complied with the ethical standards of the Human Research Ethics Committee of the Federal University of Alagoas (protocol number 65701122.8.0000.5013).

### 2.2 Viral Culture Cell Isolation and Indirect Immunofluorescence Assay (Ifa)

Serum samples from selected patients were included in the study, in which RT‐qPCR detected OROV, and the cycle threshold was < 20. The samples were inoculated onto monolayers of VERO E6 cells (CRL‐1586) and cultured in DMEM F12 medium (Gibco, USA) containing 10% heat‐inactivated fetal bovine serum (FBS), 1% l‐glutamine (Gluta‐MAX, ThermoFisher Scientific), and 1% penicillin‐streptomycin‐amphotericin B (PSA) solution. The cells were incubated at 37°C in a CO2 incubator. These cells were inoculated with serum samples. The cell cultures were monitored daily for the development of virus‐induced cytopathic effects (CPE). The aliquots were used to confirm viral isolation by RT‐qPCR and IFA.

The cells from culture pellets were fixed with a 1:1 methanol‐acetone solution for 10 min. After washing with PBS, they were permeabilized with 0.1% Triton X‐100. Samples were incubated with PBS and BSA, then treated with mouse ascitic fluid hyperimmune to anti‐OROV (1:500). The slides were washed three times with PBS and then incubated with Alexa Fluor‐conjugated anti‐mouse IgG secondary antibody (1:2000). After additional washes and staining with DAPI, the slides were mounted and examined under a fluorescence microscope.

### 2.3 Whole Genome Sequencing and Generation of Consensus Sequences

Whole genome sequencing was performed on 37 OROV‐positive samples from serum patients. Only samples with CT values of 27 or less were selected. To ensure the integrity, specificity, and reliability of the NGS results, a negative control sample (no‐template control) was included in the PCR amplification and sequencing workflow.

RNA was converted to cDNA using Luna Script RT SuperMix (5x; New England Biolabs [NEB], Ipswich, MA, USA). The generated cDNA underwent multiplex PCR sequencing using Q5 High Fidelity Hot‐Start DNA Polymerase (NEB) and a primer set designed for sequencing the three OROV segments, following the method described by Naveca and coworkers [8]. Amplicons were purified using AMPure XP beads (Beckman Coulter, Brea, CA, USA), and concentrations were determined using the Qubit dsDNA HS Assay Kit on a Qubit 4 Fluorometer (Thermo Fisher Scientific Corporation, Waltham, MA, USA). Library preparation was performed using the Ligation Sequencing Kit (SQK‐LSK109) and Native Barcoding Expansion Kit EXP‐NBD196 (Oxford Nanopore, Oxford, UK). The library was loaded onto an R10.4 flow cell and sequenced using a MinION Mk1B device. ONT MinKNOW software was used for collecting raw data. Raw files were basecalled and demultiplexed using Guppy v.6.0.1 (Oxford Nanopore Technologies). Consensus sequences were obtained through hybrid assembly using the Genome Detective online tool (https://www.genomedetective.com/). In this context, hybrid assembly refers to a computational framework that combines de novo and reference‐guided assembly methods to enhance genome completeness and accuracy. This differs from the conventional hybrid sequencing and assembly approach, which typically integrates short‐ and long‐read data from the same sample.

### 2.4 Phylogeny and Time‐Scaled Phylogenetic Tree Analysis

The S, M, and L genomic segments of OROV generated in this study were combined with corresponding segments from all published full‐length OROV sequences available in GISAID up to August 2024. Sequences with identical collection locations and dates were filtered to construct a final dataset, which included our sequences and 305 representative sequences per segment. Sequence alignment was performed using MAFFT v7.526 [18] and manually curated in AliView v1.28 [19] to remove artifacts. Maximum likelihood phylogenetic trees for each segment were constructed using IQ‐TREE v2.3.5 [20]. The TIM3 + F + I + R3 nucleotide substitution model, selected by ModelFinder [21], was used for the L and M segments, while the TPM3 + R2 model was applied to the S segment. Branch support was assessed using 1000 ultrafast bootstrap replicates and an SH‐aLRT test (1000 replicates). Trees were visualized using the ggtree R package.

To estimate the temporal and spatial dynamics of OROV in Alagoas, a time‐scaled phylogenetic tree was constructed using the Nextstrain Augur pipeline [22]. Sequences were filtered by metadata (state, municipality, and collection date), and aligned genomes from 2022 to 2025 were used to infer a maximum likelihood tree with IQ‐TREE. A time‐scaled phylogeny was generated with TreeTime using a strict molecular clock and marginal date inference. Geographic ancestral states were inferred with Augur traits at two resolutions. The dataset was then exported to Auspice, enabling interactive exploration of clades and dispersal routes, as well as the identification of the most recent common ancestor of the Alagoas sequences. This allowed us to estimate when the virus was introduced to the state.

Using the Recombination Detection Program (RDP5) [23], we analyzed the multiple sequence alignments of the three concatenated segments (S, M, and L). This allowed us to identify potential homologous recombination events involving template switching within the same genomic segment. Additionally, we investigated reassortment events, defined as the exchange of complete genomic segments among related viruses, separately. We performed segment‐specific phylogenetic reconstructions and compared the resulting tree topologies.

### 2.5 Selection Pressures

The investigation of selection pressures was performed in a first set of M gene sequences obtained in Brazil's Northeast region and a second set of sequences from all Brazilian regions: Midwest (*n* = 10), North (*n* = 173), Northeast (*n* = 111), South (*n* = 10), and Southeast (*n* = 105). The first set included 111 sequences (> 4200 nt) and the second set included 409 sequences (> 4200 nt). All sequences were obtained from human cases in 2024.

The average number of nonsynonymous substitutions (dN) and synonymous substitutions (dS) per site (dN/dS ratio) was estimated by the single likelihood ancestor counting (SLAC) [24]. SLAC estimated sites under diversifying selection (positive selection), mixed effects model of evolution (MEME) [25], and fast unconstrained Bayesian approximation (FUBAR) [26]. Sites under purifying selection (negative selection) were estimated with FUBAR and SLAC. These methods are implemented in the HYPHY platform [27] and were accessed through the DataMonkey 2.0 web server (http://www.datamonkey.org) [28]. For the MEME and SLAC methods, the confidence level was set to a p‐value of 0·05, and the FUBAR method was set to a posterior probability of 0·95.

### 2.6 Evaluation of Glycoprotein Mutations in OROV Isolates

The cDNA sequences of M polyprotein were translated into protein sequences of the viral glycoproteins Gn (1–312 aa), Nsm (313–480 aa), and Gc (481–1420 aa) using MEGAX software version 10.0.5 [29]. The alignment was performed using the MUSCLE algorithm [30]. The resolution of OROV proteins is still unavailable, except for the Gc N‐terminal region (PDB ID 6H3X), which is involved in viral fusion to the host cell. Herein, we obtained the homotrimer three‐dimensional structure of the Gc protein based on the sequence OROV‐AL29_2024‐07‐12 using Colabfold software version 1.5.2 [31] on the COSMIC2 platform (https://cosmic2.sdsc.edu:8443/gateway/login!input.action) [32]. Software basal parameters were maintained and added to the “Use templates from published PDB structures” option.

### 2.7 Epidemiological Data and Statistical Analysis

For the descriptive analysis, information on the age, sex, and municipality of residence of patients testing positive was obtained from the Laboratory Environment Manager (GAL) database, which is dedicated to health surveillance. Age categories followed the model of the Brazilian Institute of Geography and Statistics (IBGE) (https://ibge.gov.br/).

To account for differences in population size between municipalities, incidence rates were calculated and expressed as the number of confirmed OROV cases per 100,000 inhabitants. Population estimates for 2024 were obtained from official IBGE projections and used as the denominator in the calculations.

The data were tabulated and analysed using R version 4.4.1 and RStudio 2024.04.2 + 764. The tidyverse, ggplot2, geobr, ggExtra, and cowplot packages were utilized for data manipulation, statistical analysis, and visualization.

## 3 Results

**Figure 1:**
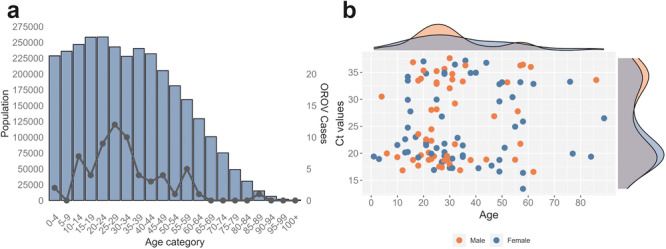
Oropouche‐positive cases distribution. (a) Distribution of the general population in the state of Alagoas by age category (blue bars), overlaid with the number of reported OROV cases as a black line. (b) Relationship between the age of individuals positive for OROV and the threshold cycle (Ct) values obtained by RT‐qPCR, differentiated by sex.

To investigate the presence of OROV in Alagoas state, we analyzed samples that tested negative for Zika, Dengue, and Chikungunya viruses using molecular diagnostics. Among the 1,316 samples examined from April to September 2024, 115 (8.74%) tested positive for OROV (Table S1). The remaining 1,201 samples (91.26%) were negative, including all those tested for MAYV. Figure 1A illustrates the age distribution of Alagoas state's population and confirmed OROV cases. The population was predominantly younger individuals aged 44 years or less. OROV incidence analysis revealed a concentrated distribution among young adults. However, there is no statistically significant association between OROV detection and age groups (Figure 1A). Regarding the gender distribution, 61 (53.04%) of the OROV‐positive patients were male, and 54 (46.96%) were female. Pearson's Chi‐squared test revealed no statistically significant association between OROV positivity and patient gender (*p*‐value > 0.05; Figure 1B, Table S1). The gender‐specific findings that stand out include the identification of 37 young women between the ages of 15 and 35 who were of reproductive age, 32.17% (37/115) of whom tested positive for OROV.

**Figure 2:**
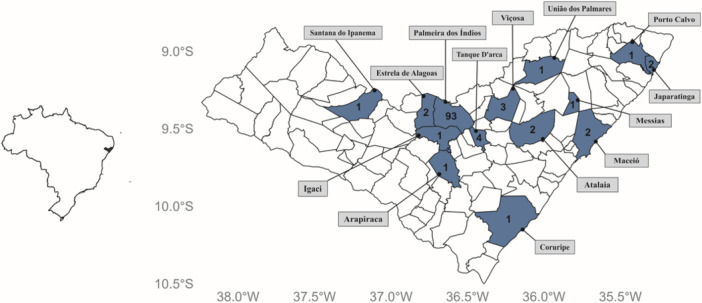
Municipalities in Alagoas with confirmed cases of Oropouche virus detected by RT‐qPCR. Internal numbers indicate the absolute number of cases.

The first case of OROV in Alagoas was identified in a male patient living in Japaratinga, a municipality on the northern coast of the state. Since then, the virus has been detected in a further 13 municipalities (Figure 2). After adjusting for population size, Palmeira dos Índios had the highest incidence rate, at around 126.4 cases per 100,000 people (93 cases among an estimated population of 73,596 in 2024). Tanque D'Arca followed with an incidence rate of 67.7 per 100,000 inhabitants (four cases), and Japaratinga with an incidence rate of 21.2 per 100,000 inhabitants (two cases). Other municipalities with lower incidence rates included Estrela de Alagoas (12.7 per 100,000) and Viçosa (12.3 per 100,000). Municipalities such as Atalaia, Maceió, Porto Calvo, Messias, Arapiraca, Coruripe, Igaci, Santana do Ipanema, and União dos Palmares each reported a single case, corresponding to incidence values of less than 10 per 100,000 inhabitants. These findings suggest a focal pattern of transmission, with disproportionate clustering in mid‐sized municipalities.

**Figure 3:**
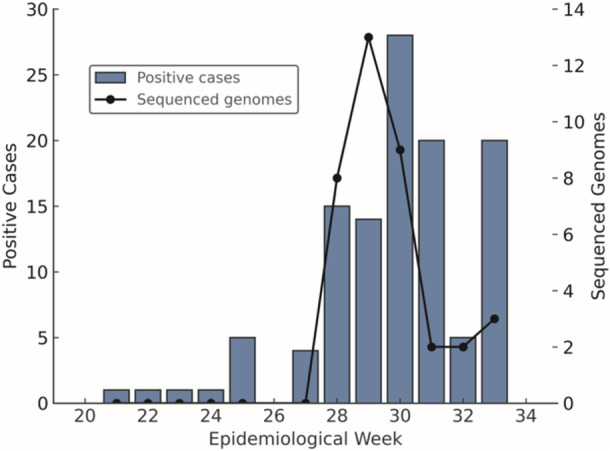
Weekly distribution of positive cases and sequenced genomes. The bar plot displays the number of positive cases per epidemiological week, while the line represents the number of genomes sequenced each week.

**Figure 4:**
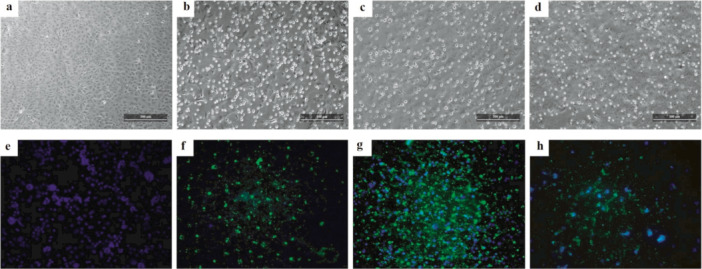
Viral cytopathic effect and IFA confirmation by Oropouche virus in human serum samples. Cytopathic effects were observed in Vero E6 cell monolayers at three days post‐infection (DPI) under 20X magnification: (A) Negative Control (NC): Vero E6 monolayer remained uninfected, showing no cytopathic effects. (B) Viral Control (VC): Vero E6 monolayer infected with the OROV strain BeAn19991, demonstrating the characteristic cytopathic effects of this strain. (C) OROV‐AL13_2024‐07‐30 wild‐type: Vero E6 monolayer infected with the wild‐type OROV strain AL13. (D) OROV‐AL17_2024‐08‐12 wild‐type: Vero E6 monolayer infected with the wild‐type OROV strain AL17. (E) Representative negative control for the immunofluorescence assay. (F) Representative positive control with OROV strain BeAn19991. (G) and (H) Immunofluorescence assay (IFA) results for the OROV wild‐type.

OROV cases in Alagoas were detected in samples collected from May to August, with the first detection on May 22 and the last on August 15 (Table S2). Positive cases were reported during epidemiological weeks 21 to 33 (Figure 3), except for week 26, which had no recorded cases. Of the 115 samples positive for the viral genome by RT‐qPCR, 14 (22.22%) underwent viral isolation attempts in Vero E6 cell culture. Culture supernatants were collected 3 days post‐infection (dpi) due to the appearance of viral cytopathic effects (CPE) in the cell monolayers, characterized by cell detachment, cytoplasmic rounding, syncytium formation, cellular debris, and plasma membrane blebbing (Figure 4). The isolation of all samples was confirmed by RT‐qPCR, with positive results for OROV, indicating isolation in the first cell culture passage (Table S3). An indirect immunofluorescence assay (IFA) was also performed on all samples subjected to isolation attempts, confirming infection of the cell monolayers.

We generated 37 new genomic sequences for each OROV segment. These sequences were obtained from cases reported during epidemiological weeks 27 to 33 (Figure 3). The average genome coverage was 89.64% for segment L, 96.20% for segment M, and 95.50% for segment S. The selected samples had an average cycle threshold (Ct) value of 17.67, with a minimum of 13.41 and a maximum of 22.57. All sequenced samples produced high‐quality data (Table S4), confirming that our selection criterion (Ct ≤ 27) was appropriate for OROV genome sequencing. The sequencing data obtained from the negative control sample showed no detectable signs of contamination, thereby confirming the integrity and reliability of the experimental procedures.

**Figure 5:**
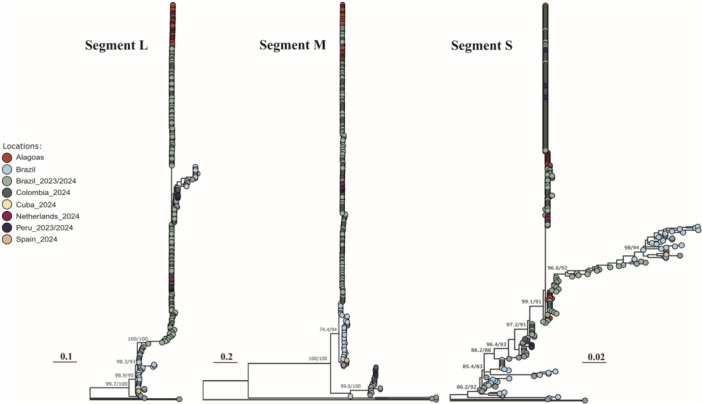
Phylogenetic analysis of the Oropouche virus. Maximum likelihood trees of 305 representative OROV genomes (L, M, and S segments), including 37 newly sequenced genomes from Alagoas (red color), were generated in this study. Tip colors represent the country of origin for the sample. The scale bar indicates the evolutionary distance in substitutions per nucleotide site. Support values for key nodes are shown as bootstrap (B) and Shimodaira‐Hasegawa (SH) scores. The Brazil_2023/2024 (green) category refers to Brazilian OROV sequences from the new reassortant lineage. The “Brazil” (light blue) category refers to Brazilian OROV sequences from before 2023.

Phylogenetic reconstruction of the L, M, and S segments from the newly generated genomes revealed a high degree of conservation with most Brazilian isolates, including sequences from the recent OROV outbreaks in Brazil (2023–2024, green; Figure 5). A well‐supported cluster was observed, grouping these sequences with previous Brazilian lineages (Brazil, blue; Figure 5), supporting the evolution of OROV from the western Amazon region of Brazil. The isolates from Alagoas (red) clustered closely with contemporary Brazilian strains, suggesting a recent common origin and indicating that these isolates likely resulted from the geographic spread of OROV within the country. The novel sequences generated in this study are available in the NCBI GenBank database under the following accession numbers: l‐segment PV335275‐PV335311, M‐segment PV254768‐PV254804, and S‐segment PV254731‐PV254767.

**Figure 6:**
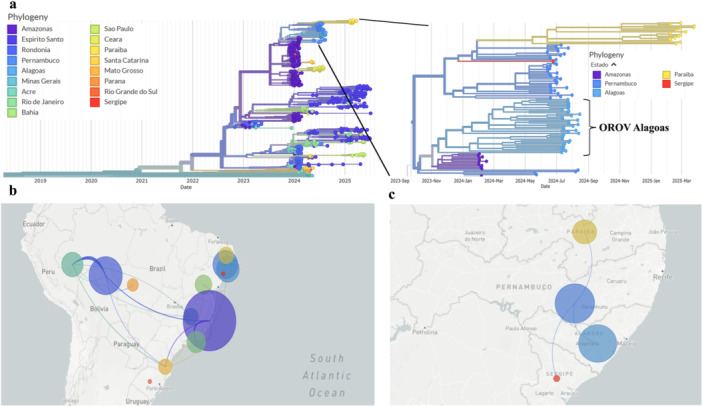
Discrete phylogeographic reconstruction of OROV circulation in Brazil (2022–2025) (a) Time‐scaled phylogeny of Brazilian OROV genomes, including sequences from Alagoas. Branches are colored by state. An expanded view of the Alagoas clade shows clustering with the northeastern states. The most recent common ancestor (MRCA) for Alagoas emerged on November 30, 2023 (90% confidence interval [CI]: March 28, 2023–February 8, 2024). (b) A nationwide model showing two independent OROV introductions into the Northeast: one from Santa Catarina to Bahia and one from Rio de Janeiro to Pernambuco. (c) Regional dispersal pathways showing the spread of the virus from Pernambuco into Alagoas, Sergipe, and Paraíba.

Discrete phylogeographic reconstruction revealed that OROV sequences from this study belonged to a Northeast Brazilian clade (Figure 6). All Alagoas genomes clustered with sequences from neighboring northeastern states, particularly Pernambuco (Figure 6a). Time‐scaled phylogeny estimated the most recent common ancestor of the Alagoas cluster emerged on November 30, 2023 (90% CI: March 28, 2023–February 8, 2024), suggesting recent introduction. Spatial diffusion analysis supported a northeastern dispersal pattern, detecting two independent OROV introductions into the Northeast: one from Santa Catarina to Bahia and another from Rio de Janeiro to Pernambuco (Figure 6b). From Pernambuco, viral movement spread into Alagoas as well as to Sergipe and Paraíba (Figure 6c). These results reveal the Alagoas outbreak is part of a regional transmission network originating from neighboring northeastern states. Unfortunately, because the majority of our sequences belonged to the municipality of Palmeira dos Índios, we were unable to verify broader OROV distribution across Alagoas. This occurred because sequences from other municipalities did not yield quality data, or positive samples with Ct values greater than 27 were not selected for sequencing.

We assessed selection pressures acting on the M gene to investigate viral evolutionary dynamics. The results are shown in Table 1. In M gene sequences from Brazil's Northeastern region obtained in 2024, FUBAR and SLAC analyses indicated 60 and 38 sites under purifying selection, respectively. FUBAR supported thirty‐eight sites, which SLAC supported. In sequences from all Brazilian regions, FUBAR and SLAC analysis indicated 104 and 66 sites under purifying selection, respectively. FUBAR supports all sites supported by SLAC. The mean ratio of nonsynonymous substitutions (dN) and synonymous substitutions (dS) rates (dN/dS) was used to identify signatures of negative (dN/dS < 1) or positive (dN/dS > 1) selection. The identified distribution rates were 0.136 and 0.173 for the sequences from Brazil's Northeast and all Brazilian regions, respectively. Sequences from the Brazilian Northeast had four and eight sites under diversifying selection, as indicated by MEME and FUBAR, respectively, and SLAC identified a single site under diversifying selection in this case. Two sites, 176 (Gn glycoprotein) and 552 (Gc glycoprotein), were identified as supported by MEME and FUBAR. For sequences from all Brazilian regions, MEME, FUBAR, and SLAC analyses indicated six, eight, and five sites under diversifying selection, respectively. The three tools support four sites: 176, 552, 981, and 982.

**Table 1:** Amino sites under diversifying and purifying selection indicated by different methods in *Orthobunyavirus oropoucheense* (OROV) sequences from Brazilian samples.

	Sites under diversifying selection			Sites under purifying selection	
	MEME^1^	FUBAR^2^	SLAC^1^	In common to FUBAR^2^ and SLAC^1^	Mean dN/dS
**Brazilian sequences from 2024**	176; 545; 552; 981; 982; 1342	61; 66; 176; 552; 846; 981; 982; 1342	66; 176; 552; 981; 982	7; 15; 57; 63; 72; 83; 86; 92; 113; 136; 140; 144; 153; 159; 162; 165; 167; 181; 194; 239; 266; *389*; *415*; *455*; 488; 516; 672; 674; 681; 730; 733; 762; 844; 845; 880; 937; 1041; 1048; 1051; 1060; 1063; 1082; 1102; 1109; 1119; 1122; 1126; 1134; 1141; 1145; 1156; 1158; 1168; 1254; 1267; 1275; 1290; 1297; 1307; 1310; 1317; 1322; 1331; 1345; 1348	0·173
**Northeast Brazil sequences from 2024**	176; 552; 980; 1325	12; 61; 66; 176; 552; 732; 982; 1342	—	7; 15; 63; 72; 86; 92; 159; 162; 165; 167; 516; 733; 762; 1134; 1141; 1290; 1302; 1310; 1348; 1349	0·136

Underlined amino acid positions: Gn glycoprotein; Amino acid positions in italic: Nsm glycoprotein. Regular font: Gc glycoprotein.

1 *p*‐value 0.05.

2 Posterior probability of 0.95.

**Figure 7:**
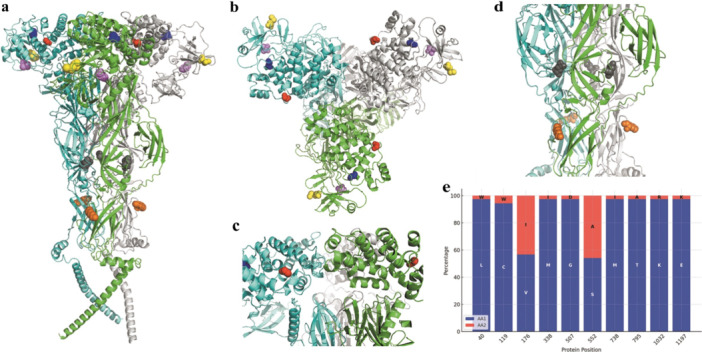
Locations of amino acid residue mutations in the homotrimer model of the Gc glycoprotein. The mutation regions are marked with different colors: red (G507D), blue (S552A), yellow (M738I), cyan (I795A), orange (K1032R), and gray (E1197 K). (A) Full view of the model. (B) View of the upper region (head) of the homotrimer. (C) View of the upper region of the monomer, and (D) View of the medial region of the homotrimer. L: leucine, W: tryptophan, C: cysteine, V: valine, I: isoleucine, M: methionine, G: glycine, D: aspartic acid, S: serine, T: threonine, K: lysine, R: arginine, E: glutamic acid. (E) Percentage of mutations in glycoproteins among the evaluated isolates. Protein positions were based on the sequence of the M polyprotein (Gn; 1–312, Nsm; 313–480, and Gc; 481–1420). L: leucine, W: tryptophan, C: cysteine, V: valine, I: isoleucine, M: methionine, G: glycine, D: aspartic acid, S: serine, T: threonine, K: lysine, R: arginine, E: glutamic acid.

The 37 analyzed sequences contained amino acid mutations in 10 glycoprotein regions. Gn, Nsm, and Gc had 3, 1, and 6 mutations, respectively. Based on the tridimensional Gc glycoprotein structure, mutations at amino acid positions 507, 552, 738, and 795 were in a region that may play a role in host‐cell interactions (Figure 7).

## 4 Discussion

Information on arboviruses is vital for public health monitoring. The symptoms of Oropouche fever are similar to those of Dengue and Chikungunya, which makes syndromic detection difficult [33]. In response to the increase in OROV cases across Brazil, including regions beyond the Amazon, the Coordination of Public Health Laboratories of the Ministry of Health (CGLAB/MS) implemented a strategy in 2024 for distributing a molecular diagnostic protocol using RT‐qPCR to Brazil's central public health laboratories, aiming to assess the extent of viral dissemination nationally. In Alagoas specifically, all serum samples that are negative for Zika, Dengue, and Chikungunya must be tested for OROV and MAYV. This change represents a critical shift from targeted surveillance, which will likely lead to an increase in reported cases of these emerging viruses and provide a more accurate picture of their circulation beyond the Amazon region.

This study reports OROV detection in Alagoas, with 115 cases recorded. Brazil confirmed 7931 OROV cases, including two deaths, by Epidemiological Week 35 of 2024. The first confirmed case of Oropouche fever in Alagoas was reported on May 22, 2024, in a male patient from Japaratinga, a town on the state's northern coast. This area is characterized by intense population mobility driven by tourism [34] and its proximity to Pernambuco, which had already reported 92 confirmed cases by the time Alagoas's first case was detected. After the initial case, new OROV cases were reported in other municipalities, particularly in Palmeiras dos Índios. The high number of cases may be related to the traditional and historical habitation of indigenous communities, whose sociocultural dynamics favor viral circulations [35], as well as to environmental factors, since the region is a significant area of banana cultivation, which has been positively correlated with OROV cases [16]. In addition, the presence of *Culicoides insignis* in Alagoas, a potential vector of the virus [36], highlights the role of local ecological conditions in transmission.

Our study also found cases in young women of reproductive age (15 to 35), with 32,17% (37/115) being OROV‐positive. This finding is concerning in public health, as studies have discussed the potential vertical transmission of OROV, which may significantly impact fetal development [37]. Increased surveillance and attention to pregnant women are essential. Reports of fetal deaths and vertical transmission of OROV have been confirmed in various Brazilian regions [38], although not yet in Alagoas.

Understanding arbovirus circulation and the factors predisposing to dissemination risk is essential for monitoring and mapping affected areas. Early diagnosis supports data collection, facilitating epidemiological and clinical studies. In Alagoas, OROV was first identified in May 2024, despite neighboring states, Pernambuco and Bahia, having diagnosed the virus months earlier [39]. This diagnostic delay may reflect either a late introduction of the virus in Alagoas or limited surveillance sensitivity, highlighting the need for enhanced, timely arbovirus monitoring strategies across different states. Early diagnosis also enables effective surveillance and timely healthcare, improving the prognosis and treatment of arbovirus‐infected patients [40], a critical goal in Brazil. Alagoas is also known for the circulation of other arboviruses, such as Dengue, Zika, and Chikungunya, which, like OROV, are considered dengue‐like viruses due to their similar symptoms: arthralgic and myalgic fever, followed by neurological and dermatological complications. This symptom overlap complicates differential diagnosis, underscoring the importance of biomolecular detection.

Phylogenetic analysis of the three OROV genome segments (L, M, and S) obtained in this study, along with reference sequences, revealed a monophyletic clade comprising sequences from recent OROV outbreaks in Brazil. This finding confirms the current circulation of a viral lineage previously reported in other Brazilian regions [39]. The high sequence similarity between Alagoas strains and those from recent national outbreaks suggests limited genetic divergence, consistent with recent viral expansion. Although genetic changes are common in segmented viruses, our sequences showed no evidence of homologous recombination or reassortment. Our sequences originate from a recently circulating reassortant lineage in Brazil, which contains M segments from viruses detected in the eastern Amazon region and L and S segments from viruses found in Peru, Colombia, and Ecuador [41]. These results support OROV's geographic dispersion across Brazil, particularly in areas with no history of endemicity or infection risk.

Recent genomic analyses have demonstrated the introduction of the BR‐2015‐2024 lineage into Colombia, where it co‐circulates with a previously established OROV lineage (OROV PE/CO/EC‐2008‐2021), indicating that this variant is spreading beyond Brazil throughout the Amazon Basin [42]. The simultaneous circulation of distinct lineages increases the likelihood of reassortment and may generate viruses with altered transmissibility or pathogenicity. This scenario underscores the importance of continuous genomic surveillance and cross‐border monitoring to detect new introductions, characterize emerging variants, and prevent the wider dissemination of OROV across the Americas.

Residues 176 (Gn) and 552 (Gc), which showed the most homogeneous frequencies of amino acid mutations among the sequences obtained in this study [Figure 5], were also identified as under diversifying selection pressures based on both Brazilian and Northeast Brazilian sequences. Other mutations identified in this study were not under selection pressure. Additionally, under diversifying selection, residue 61 (Gn) has been identified as an emerging site of the non‐synonymous substitution V61F, which may have emerged between December 2023 and April 2024 in Brazilian OROV sequences [43]. However, all 37 sequences obtained from May to August 2024 presented V61. In line with our findings, sites 66 and 86 (Gn) were also identified as undergoing diversifying and purifying selection, respectively, in another study that analyzed OROV samples from Trinidad and Tobago, Panama, Peru, and Brazil from the 1950s to the late 2000s [41].

The Gn and Gc glycoproteins play an important role in OROV architecture. Studies on crystallography and comparison of protein sequences among species have demonstrated that these proteins may form trimeric structures [44]. Thus, these glycoproteins may represent crucial targets for antigenic recognition and cell interactions, highlighting the importance of investigating variations in their amino acid residues. Our data demonstrate two and five amino acid modifications in Gn and Gc, respectively, which alter their chemical properties. In Gn, at position 40, leucine (an apolar aliphatic residue) was replaced by tryptophan. In position 119, cysteine (a polar residue) was also substituted by tryptophan, introducing aromatic properties in both cases. In Gc, modifications were observed in 507 residues from glycine to aspartic acid (negatively charged); at position 552, serine was replaced by alanine (apolar, aliphatic); at position 738, methionine was replaced by isoleucine; at position 795, threonine was replaced by alanine; and at position 1197, glutamic acid (negatively charged) was replaced by lysine (positively charged). Given this, modifications in the residues of glycoproteins may represent significant changes in the immune response and virus‐cell interactions.

This study has both inherent limitations and noteworthy strengths. One of its main advantages is being the first report of Oropouche virus circulation in Alagoas, Brazil, supported by extensive molecular, virological, and genomic evidence, including the generation of 37 nearly complete genome sequences, which enhances the originality and robustness of the findings. Additionally, the epidemiological analysis provides valuable insights, particularly regarding the identification of reproductive‐age women as a potentially vulnerable group, which has important public health implications. However, certain limitations must be acknowledged. The study is primarily descriptive and lacks clinical outcome data. Moreover, the absence of serological investigations precludes the evaluation of prior or asymptomatic infections. Ultimately, despite the study highlighting the significance of enhanced surveillance, incorporating more detailed recommendations for public health officials would enhance its practical applicability.

## 5 Conclusion

We confirmed the first case of OROV in Alagoas through molecular detection in samples that tested negative for other arboviruses. This finding corroborates recent findings from various studies, which have shown a shift in the geographic distribution of OROV cases, previously concentrated in northern Brazil, indicating an expansion of its transmission area. This highlights the need to intensify epidemiological surveillance in other regions with similar symptoms but limited testing for this arbovirus.

Herein, we recommend implementing genomic surveillance strategies nationwide to enhance monitoring and understanding of viral circulation, providing essential data for public health decision‐making. This approach will enable rapid detection of potential outbreaks, improve understanding of transmission dynamics, and support the development of targeted interventions to control the virus's spread. Our findings underscore the need for coordinated actions among researchers, health authorities, and the community to address the emerging challenges posed by OROV in Brazil.

## Conflicts of Interest

The authors declare no conflicts of interest.

## Supporting information

S5 ‐ Supporting material GISAID.

**Table S1:** Comparison of demographic characteristics between OROV RT‐qPCR‐positive and negative patients.

**Table S2:** Epidemiological information of Oropouche‐positive patients detected in Alagoas.

**Table S3:** Cell culture supernatant (1st Passage) processed by RT‐qPCR and Indirect Immunofluorescence.

**Table S4:** Metrics of Alagoas OROV sequences.

## Data Availability

The data that support the findings of this study are available from the corresponding author upon reasonable request.

## References

[1] J. F. Travassos Da Rosa , W. M. De Souza , F. P. Pinheiro , et al., “Oropouche Virus: Clinical, Epidemiological, and Molecular Aspects of a Neglected Orthobunyavirus,” American Society of Tropical Medicine and Hygiene 96, no. 5 (2017): 1019–1030, 10.4269/ajtmh.16-0672. PMC5417190 28167595

[2] F. P. Pinheiro , M. Pinheiro , G. Bensabath , O. R. Causey , and R. E. Shope , “Epidemia De Vírus Oropouche Em Belém. *Revista Do* ,” Serviço Especial de Saúde Pública 12 (1962): 15–23.

[3] P. R. Martins‐Filho , T. A. Carvalho , and C. A. dos Santos , “Spatiotemporal Epidemiology of Oropouche Fever, Brazil,” 2015–2024, Emerging Infectious Diseases 30, no. 10 (2024): 2196–2198, 10.3201/eid3010.241088. 39213265 PMC11431927

[4] Brasil . Ministério da Saúde. Secretaria de Vigilância em Saúde e Ambiente. Oropouche. 2024 [cited 2024 Sep 6], https://www.gov.br/saude/pt-br/assuntos/saude-de-a-a-z/o/oropouche/painel-epidemiologico.

[5] R. M. Elliott , “Orthobunyaviruses: Recent Genetic and Structural Insights,” Nature Reviews Microbiology 12 (2014): 673–685, 10.1038/nrmicro3332. 25198140

[6] H. R. Hughes , S. Adkins , S. Alkhovskiy , et al., “ICTV Virus Taxonomy Profile: Peribunyaviridae,” Journal of General Virology 101 (2020): 1–2, 10.1099/jgv.0.001365. 31846417 PMC7414433

[7] K. Gunter , D. Omoga , J. M. Bowen , et al., “A Reporter Oropouche Virus Expressing Zsgreen From the M Segment Enables Pathogenesis Studies in Mice,” Journal of Virology 98, no. 9 (2024): e00893‐24, 10.1128/jvi.00893-24. 39194249 PMC11406970

[8] F. G. Naveca , T. A. P. Almeida , V. Souza , et al., “Human Outbreaks of a Novel Reassortant Oropouche Virus in the Brazilian Amazon Region,” Nature Medicine 30 (2024): 3509–3521, 10.1038/s41591-024-03300-3. 39293488

[9] K. LaTourrette and H. Garcia‐Ruiz , “Determinants of Virus Variation, Evolution, and Host Adaptation,” Pathogens 11, no. 9 (2022): 1039, 10.3390/pathogens11091039. 36145471 PMC9501407

[10] S. L. P. Scroggs , J. Gutierrez , L. M. Reister‐Hendricks , K. B. Gunter , N. L. Tilston , and B. L. McGregor , “Enhanced Infection and Transmission of the 2022–2024 Oropouche Virus Strain in the North American Biting Midge Culicoides Sonorensis,” Scientific Reports 15 (2025): 27368, 10.1038/s41598-025-11337-8. 40717076 PMC12301478

[11] M. K. Merchant , J. de Paula Souza , S. Abdelkarim , et al., “Protein‐Based Tools for the Detection and Characterisation of Oropouche Virus Infection,” EMBO Molecular Medicine 17 (2025): 2462–2482, 10.1038/s44321-025-00291-7. 40790101 PMC12423313

[12] Y. Zhang , X. Liu , Z. Wu , et al., “Oropouche Virus: A Neglected Global Arboviral Threat,” Virus Research 341 (2024): 199318, 10.1016/j.virusres.2024.199318. 38224842 PMC10827532

[13] J. F. Gómez‐Marín , J. A. Usme , G. Parra‐Henao , M. Martínez‐Gutiérrez , and J. Ruiz‐Sáenz , “Oropouche Virus Entomovirology and Surveillance: Where Are We and Where are We Headed?,” Acta Tropica 271 (2025): 107890, 10.1016/j.actatropica.2025.107890. 41176045

[14] C. Lorenz , T. S. de Azevedo, , M. A. M. Sallum , and F. Chiaravalloti‐Neto , “Oropouche Fever Outbreak in Brazil: Key Factors Behind the Largest Epidemic in History,” PLoS One 20, no. 7 (2025): e0327845, 10.1371/journal.pone.0327845. 40720414 PMC12303302

[15] P. R. Martins‐Filho , T. A. Carvalho , and C. A. dos Santos , “Oropouche Fever: Reports of Vertical Transmission and Deaths in Brazil,” Lancet Infectious Diseases 24, no. 11 (2024): e662–e663, 10.1016/S1473-3099(24)00557-7. 39182500

[16] T. Gräf , E. Delatorre , C. do Nascimento Ferreira , et al., “Expansion of Oropouche Virus in Non‐Endemic Brazilian Regions: Analysis of Genomic Characterisation and Ecological Drivers,” Lancet Infectious Diseases 25, no. 4 (2025): 379–389, 10.1016/S1473-3099(24)00687-X. 39557055

[17] F. G. Naveca , V. A. Nascimento , V. C. Souza , B. T. D. Nunes , D. S. G. Rodrigues , and P. F. C. Vasconcelos , “Multiplexed Reverse Transcription Real‐Time Polymerase Chain Reaction for Simultaneous Detection of Mayaro, Oropouche, and Oropouche‐Like Viruses,” Memórias do Instituto Oswaldo Cruz 112, no. 7 (2017): 510–513, 10.1590/0074-02760160062. 28591313 PMC5452489

[18] K. Katoh and D. M. Standley , “MAFFT Multiple Sequence Alignment Software Version 7: Improvements in Performance and Usability,” Molecular Biology and Evolution 30, no. 4 (2013): 772–780, 10.1093/molbev/mst010. 23329690 PMC3603318

[19] A. Larsson , “AliView: A Fast and Lightweight Alignment Viewer and Editor for Large Datasets,” Bioinformatics 30, no. 22 (2014): 3276–3278, 10.1093/bioinformatics/btu531. 25095880 PMC4221126

[20] B. Q. Minh , H. A. Schmidt , O. Chernomor , et al., “IQ‐tree 2: New Models and Efficient Methods for Phylogenetic Inference in the Genomic Era,” Molecular Biology and Evolution 37, no. 5 (2020): 1530–1534, 10.1093/molbev/msaa015. 32011700 PMC7182206

[21] S. Kalyaanamoorthy , B. Q. Minh , T. K. F. Wong , A. von Haeseler , and L. S. Jermiin , “Modelfinder: Fast Model Selection for Accurate Phylogenetic Estimates,” Nature Methods 14, no. 6 (2017): 587–589, 10.1038/nmeth.4285. 28481363 PMC5453245

[22] J. Hadfield , C. Megill , S. M. Bell , et al., “Nextstrain: Real‐Time Tracking of Pathogen Evolution,” Bioinformatics 34, no. 23 (2018): 4121–4123, 10.1093/bioinformatics/bty407. 29790939 PMC6247931

[23] D. P. Martin , A. Varsani , P. Roumagnac , et al., “RDP5: a Computer Program for Analyzing Recombination in, and Removing Signals of Recombination from, Nucleotide Sequence Datasets,” Virus Evolution 7, no. 1 (2020): veaa087, 10.1093/ve/veaa087. 33936774 PMC8062008

[24] S. L. Kosakovsky Pond and S. D. W. Frost , “Not so Different After All: A Comparison of Methods for Detecting Amino Acid Sites Under Selection,” Molecular Biology and Evolution 22, no. 5 (2005): 1208–1222, 10.1093/molbev/msi105. 15703242

[25] B. Murrell , J. O. Wertheim , S. Moola , T. Weighill , K. Scheffler , and S. L. Kosakovsky Pond , “Detecting Individual Sites Subject to Episodic Diversifying Selection,” PLoS Genetics 8, no. 7 (2012): e1002764, 10.1371/journal.pgen.1002764. 22807683 PMC3395634

[26] B. Murrell , S. Moola , A. Mabona , et al., “FUBAR: A Fast, Unconstrained Bayesian Approximation for Inferring Selection,” Molecular Biology and Evolution 30, no. 5 (2013): 1196–1205, 10.1093/molbev/mst030. 23420840 PMC3670733

[27] S. L. Kosakovsky Pond , A. F. Y. Poon , R. Velazquez , et al., “HyPhy 2.5 A Customizable Platform for Evolutionary Hypothesis Testing Using Phylogenies,” Molecular Biology and Evolution 37, no. 1 (2019): 295–299, 10.1093/molbev/msz197. PMC8204705 31504749

[28] S. Weaver , S. D. Shank , S. J. Spielman , M. Li , S. V. Muse , and S. L. Kosakovsky Pond , “Datamonkey 2.0: A Modern Web Application for Characterizing Selective and Other Evolutionary Processes,” Molecular Biology and Evolution 35, no. 3 (2018): 773–777, 10.1093/molbev/msx335. 29301006 PMC5850112

[29] S. Kumar , G. Stecher , M. Li , C. Knyaz , and K. Tamura , “MEGA X: Molecular Evolutionary Genetics Analysis Across Computing Platforms,” Molecular Biology and Evolution 35, no. 6 (2018): 1547–1549, 10.1093/molbev/msy096. 29722887 PMC5967553

[30] R. C. Edgar , “MUSCLE: A Multiple Sequence Alignment Method With Reduced Time and Space Complexity,” BMC Bioinformatics 5 (2004): 113, 10.1186/1471-2105-5-113. 15318951 PMC517706

[31] M. Mirdita , K. Schütze , Y. Moriwaki , L. Heo , S. Ovchinnikov , and M. Steinegger , “Colabfold: Making Protein Folding Accessible to All,” Nature Methods 19 (2022): 679–682, 10.1038/s41592-022-01488-1. 35637307 PMC9184281

[32] M. M. A. Cianfrocco , C. Wong‐Barnum , R. Youn , A. Wagner , and Leschziner , “COSMIC2: A Science Gateway for Cryo‐Electron Microscopy Structure Determination. In: PEARC ‘17,” Practice and Experience in Advanced Research Computing 2017; New Orleans; 2017 Jul 9–13. Abstract 22 (New York (NY): Association for Computing Machinery, 2017), 10.1145/3093338.3093390.

[33] A. N. Desai , A. Otter , M. Koopmans , et al., “Oropouche Virus: A Re‐Emerging Arbovirus of Clinical Significance,” IJID regions 13 (2024): 100456, 10.1016/j.ijregi.2024.100456. 39507390 PMC11539570

[34] B. R. Embratur . Briefing para Alagoas 2024. Brasília: Embratur; 2024, http://dados.embratur.com.br.

[35] C. E. Coimbra, Jr. , R. V. Santos , and A. M. Cardoso , “Povos Indígenas No Processo Saúde‐Doença,” Vigilância alimentar e nutricional para a Saúde Indígena. 22th ed. Rio de Janeiro: Fiocruz 1 (2007): 47–74, 10.7476/9788575415870.

[36] R. R. S. Rios , M. C. A. Santarém , K. A. L. Ribeiro Júnior , et al., “Culicoides Insignis Lutz, 1913 (Diptera: Ceratopogonidae) Biting Midges in Northeast of Brazil,” Insects 12, no. 4 (2021): 366, 10.3390/insects12040366. 33924170 PMC8074382

[37] R. Sah , S. Srivastava , R. Mehta , et al., “Oropouche Fever Fatalities and Vertical Transmission in South America: Implications of a Potential New Mode of Transmission,” Lancet regional health. Americas 38 (2024): e100896, 10.1016/j.lana.2024.100896. PMC11459618 39381084

[38] C. Garcia Filho , A. S. Lima Neto , A. M. P. C. Maia , et al., “A Case of Vertical Transmission of Oropouche Virus in Brazil,” New England Journal of Medicine 391, no. 21 (2024): 2055–2057, 10.1056/NEJMc2412812. 39476360

[39] E. A. N. Azevedo , A. F. Silva , V. G. Silva , et al., “Genomic and Phenotypic Characterization of the Oropouche Virus Strain Implicated in the 2022–24 Large‐Scale Outbreak in Brazil,” Journal of Medical Virology 96, no. 10 (2024): e70012, 10.1002/jmv.70012. 39415323

[40] M. A. Files , C. A. Hansen , V. C. Herrera , et al., “Baseline Mapping of Oropouche Virology, Epidemiology, Therapeutics, and Vaccine Research and Development,” NPJ Vaccines 7 (2022): 38, 10.1038/s41541-022-00456-2. 35301331 PMC8931169

[41] B. Gutierrez , E. L. Wise , S. T. Pullan , et al., “Evolutionary Dynamics of Oropouche Virus in South America,” Journal of Virology 94, no. 5 (2020): e10.1128, 10.1128/jvi.01127-19. PMC7022353 31801869

[42] J. Usuga , D. Limonta , L. S. Perez‐Restrepo , et al., “Co‐Circulation of 2 Oropouche Virus Lineages, Amazon Basin, Colombia, 2024,” Emerging Infectious Diseases 30, no. 11 (2024): 2375–2380, 10.3201/eid3011.240405. 39356574 PMC11521167

[43] F. R. R. Moreira , J. V. R. Dutra , A. H. B. de Carvalho , et al., “Oropouche Virus Genomic Surveillance in Brazil,” Lancet Infectious Diseases 24, no. 11 (2024): e664–e666, 10.1016/S1473-3099(24)00558-9. 39208828

[44] J. Hellert , A. Aebischer , A. Haouz , et al., “Structure, Function, and Evolution of the Orthobunyavirus Membrane Fusion Glycoprotein,” Cell Reports 42, no. 3 (2023): 112142, 10.1016/j.celrep.2023.112142. 36827185

