# Supplementary material for: Arrival of Oropouche Virus in a Nonendemic Area in Northeastern Brazil, 2024
Source: J Med Virol. 2025 Dec 29;98(1):e70780. doi: 10.1002/jmv.70780 (PMC12746540; doi:10.1002/jmv.70780)
Supplement: Supplementary file 1 — S5 ‐ Supporting material GISAID. [file JMV-98-e70780-s002.docx]

SUPPLEMENTARY MATERIAL

**Data Availability**

GISAID Identifier: EPI_SET_251103od

doi: <https://doi.org/10.55876/gis8.251103od>

All genome sequences and associated metadata supporting the findings of this study are publicly available on gisaid.org and can be accessed through the persistent digital object identifier <https://doi.org/10.55876/gis8.251103od>. In addition to the minted DOI, GISAID also aggregates GISAID accession numbers (EPI_ISL_IDs) via the corresponding EPI_SET_251103od identifier to facilitate both acknowledging all data contributors and directly retrieving the underlying data used in this study from GISAID.

**Data Snapshot**

- EPI_SET_251103od comprises 1,409 GISAID accession numbers.
- The dataset includes all sequences available up to August 25, 2025.
- Data were collected in 17 countries and territories.
