# Supplementary material for: Arrival of Oropouche Virus in a Nonendemic Area in Northeastern Brazil, 2024
Source: J Med Virol. 2025 Dec 29;98(1):e70780. doi: 10.1002/jmv.70780 (PMC12746540; doi:10.1002/jmv.70780)
Supplement: Supplementary file 2 — Table S1: Comparison of demographic characteristics between OROV RT‐qPCR‐positive and negative patients. [file JMV-98-e70780-s004.docx]

**Table S1 - Comparison of demographic characteristics between OROV RT-qPCR-positive and negative patients. Absolute counts and percentages are shown for each category. P-values were calculated using the chi-square test (sex) and the Mann-Whitney U test (age). Percentages represent proportions within each diagnostic group.**

| Variable | OROV positive | OROV negative | p-value |
| --- | --- | --- | --- |
| Male (%) | 61 (53%) | 529 (44%) | 0.08 |
| Female (%) | 54 (47%) | 672 (56%) | — |
| Mean age (years) | 33.1 | 30.1 | 0.06 |
| Age group <1 year (%) | 0 (0%) | 0 (0%) | — |
| Age group 1–14 (%) | 14 (12%) | 242 (20%) | — |
| Age group 15–19 (%) | 10 (9%) | 135 (11%) | — |
| Age group 20–39 (%) | 58 (50%) | 495 (41%) | — |
| Age group 40–59 (%) | 25 (22%) | 235 (20%) | — |
| Age group 60–69 (%) | 3 (3%) | 52 (4%) | — |
| Age group 70–79 (%) | 2 (2%) | 30 (2%) | — |
| Age group 80–89 (%) | 3 (3%) | 10 (1%) | — |
| Age group 90+ (%) | 0 (0%) | 2 (0%) | — |
