# Supplementary material for: Arrival of Oropouche Virus in a Nonendemic Area in Northeastern Brazil, 2024
Source: J Med Virol. 2025 Dec 29;98(1):e70780. doi: 10.1002/jmv.70780 (PMC12746540; doi:10.1002/jmv.70780)
Supplement: Supplementary file 3 — Table S2: Epidemiological information of Oropouche‐positive patients detected in Alagoas. [file JMV-98-e70780-s003.docx]

**Table S2 - Epidemiological information of Oropouche-positive patients detected in Alagoas**

| **Collection_date** | **EW*** | **Age** | **Gender** | **Municipality** | **Ct_value**** | **Viral_load^#^** |
| --- | --- | --- | --- | --- | --- | --- |
| 2024-05-22 | 21 | 55 | Male | Japaratinga | 24·93 | high |
| 2024-05-29 | 22 | 32 | Female | Japaratinga | 29·23 | moderate |
| 2024-06-05 | 23 | 23 | Female | Tanque D'arca | 28·05 | moderate |
| 2024-06-15 | 24 | 24 | Male | Palmeira Dos Índios | 16·9 | high |
| 2024-06-16 | 25 | 57 | Female | Estrela De Alagoas | 36·28 | low |
| 2024-06-16 | 25 | 62 | Male | Palmeira Dos Índios | 32·86 | low |
| 2024-06-20 | 25 | 19 | Female | Porto Calvo | 19·63 | high |
| 2024-06-21 | 25 | 36 | Male | Messias | 37·19 | low |
| 2024-06-22 | 25 | 40 | Female | Atalaia | 34·95 | low |
| 2024-07-03 | 27 | 34 | Female | Tanque D'arca | 16·85 | high |
| 2024-07-03 | 27 | 27 | Female | Tanque D'arca | 17·39 | high |
| 2024-07-04 | 27 | 30 | Female | Arapiraca | 37·63 | low |
| 2024-07-06 | 27 | 25 | Male | Palmeira Dos Índios | 20·97 | high |
| 2024-07-07 | 28 | 52 | Female | Palmeira Dos Índios | 33·15 | low |
| 2024-07-07 | 28 | 36 | Female | Palmeira Dos Índios | 36·29 | low |
| 2024-07-07 | 28 | 14 | Male | Palmeira Dos Índios | 34·19 | low |
| 2024-07-07 | 28 | 14 | Male | Palmeira Dos Índios | 29·91 | moderate |
| 2024-07-07 | 28 | 35 | Female | Palmeira Dos Índios | 33·32 | low |
| 2024-07-07 | 28 | 12 | Female | Tanque D'arca | 16·83 | high |
| 2024-07-09 | 28 | 13 | Male | Palmeira Dos Índios | 22·6 | high |
| 2024-07-09 | 28 | 20 | Male | Viçosa | 37·04 | low |
| 2024-07-10 | 28 | 35 | Male | Palmeira Dos Índios | 19·33 | high |
| 2024-07-10 | 28 | 25 | Female | Palmeira Dos Índios | 35·23 | low |
| 2024-07-11 | 28 | 14 | Male | Atalaia | 20·24 | high |
| 2024-07-12 | 28 | 46 | Male | Palmeira Dos Índios | 17·71 | high |
| 2024-07-12 | 28 | 22 | Female | Palmeira Dos Índios | 20·11 | high |
| 2024-07-13 | 28 | 27 | Male | Palmeira Dos Índios | 18·26 | high |
| 2024-07-13 | 28 | 18 | Female | Palmeira Dos Índios | 17·71 | high |
| 2024-07-15 | 29 | 22 | Female | Palmeira Dos Índios | 18·78 | high |
| 2024-07-15 | 29 | 30 | Male | Palmeira Dos Índios | 17·29 | high |
| 2024-07-15 | 29 | 29 | Female | Palmeira Dos Índios | 18·87 | high |
| 2024-07-15 | 29 | 25 | Female | Palmeira Dos Índios | 32·87 | low |
| 2024-07-15 | 29 | 23 | Female | Palmeira Dos Índios | 22·32 | high |
| 2024-07-16 | 29 | 18 | Female | Coruripe | 33·48 | low |
| 2024-07-17 | 29 | 13 | Male | Palmeira Dos Índios | 20·14 | high |
| 2024-07-17 | 29 | 28 | Male | Palmeira Dos Índios | 21·78 | high |
| 2024-07-17 | 29 | 58 | Male | Viçosa | 25·89 | moderate |
| 2024-07-18 | 29 | 19 | Female | Palmeira Dos Índios | 33·83 | low |
| 2024-07-18 | 29 | 49 | Male | Palmeira Dos Índios | 29·61 | moderate |
| 2024-07-19 | 29 | 58 | Male | Palmeira Dos Índios | 13·41 | high |
| 2024-07-19 | 29 | 27 | Male | Viçosa | 26·82 | moderate |
| 2024-07-19 | 29 | 86 | Female | Estrela De Alagoas | 33·58 | low |
| 2024-07-22 | 30 | 14 | Male | Palmeira Dos Índios | 33·47 | low |
| 2024-07-22 | 30 | 57 | Male | Palmeira Dos Índios | 33·18 | low |
| 2024-07-22 | 30 | 31 | Female | Palmeira Dos Índios | 33·68 | low |
| 2024-07-22 | 30 | 25 | Male | Palmeira Dos Índios | 29·96 | moderate |
| 2024-07-23 | 30 | 40 | Male | Palmeira Dos Índios | 34·93 | low |
| 2024-07-23 | 30 | 32 | Female | Palmeira Dos Índios | 21·68 | high |
| 2024-07-23 | 30 | 23 | Female | Palmeira Dos Índios | 18·93 | high |
| 2024-07-23 | 30 | 21 | Female | Palmeira Dos Índios | 22·57 | high |
| 2024-07-23 | 30 | 25 | Female | Palmeira Dos Índios | 33·14 | low |
| 2024-07-23 | 30 | 30 | Female | Palmeira Dos Índios | 17·96 | high |
| 2024-07-24 | 30 | 27 | Male | Palmeira Dos Índios | 18·4 | high |
| 2024-07-24 | 30 | 4 | Female | Palmeira Dos Índios | 30·51 | low |
| 2024-07-24 | 30 | 41 | Male | Palmeira Dos Índios | 17·23 | high |
| 2024-07-24 | 30 | 31 | Male | Palmeira Dos Índios | 17·26 | high |
| 2024-07-25 | 30 | 3 | Male | Palmeira Dos Índios | 18·93 | high |
| 2024-07-25 | 30 | 31 | Male | Santana Do Ipanema | 19·01 | high |
| 2024-07-25 | 30 | 1 | Male | Palmeira Dos Índios | 19·4 | high |
| 2024-07-25 | 30 | 28 | Male | Palmeira Dos Índios | 18·59 | high |
| 2024-07-25 | 30 | 49 | Male | Palmeira Dos Índios | 18·76 | high |
| 2024-07-25 | 30 | 21 | Male | Palmeira Dos Índios | 21·93 | high |
| 2024-07-25 | 30 | 84 | Male | Palmeira Dos Índios | 19·37 | high |
| 2024-07-25 | 30 | 49 | Male | Palmeira Dos Índios | 16·61 | high |
| 2024-07-25 | 30 | 33 | Male | Palmeira Dos Índios | 22·86 | high |
| 2024-07-25 | 30 | 26 | Female | Palmeira Dos Índios | 17.53 | high |
| 2024-07-26 | 30 | 6 | Female | Palmeira Dos Índios | 19·97 | high |
| 2024-07-27 | 30 | 61 | Female | Palmeira Dos Índios | 36·01 | low |
| 2024-07-27 | 30 | 21 | Male | Palmeira Dos Índios | 21·49 | high |
| 2024-07-27 | 30 | 23 | Male | Palmeira Dos Índios | 19·92 | high |
| 2024-07-28 | 31 | 50 | Male | Palmeira Dos Índios | 33·16 | low |
| 2024-07-29 | 31 | 89 | Male | Palmeira Dos Índios | 26·49 | moderate |
| 2024-07-29 | 31 | 18 | Male | Palmeira Dos Índios | 19·23 | high |
| 2024-07-29 | 31 | 15 | Male | Palmeira Dos Índios | 27·76 | moderate |
| 2024-07-29 | 31 | 17 | Male | Palmeira Dos Índios | 23·01 | high |
| 2024-07-29 | 31 | 10 | Female | Palmeira Dos Índios | 19·28 | high |
| 2024-07-30 | 31 | 35 | Male | Palmeira Dos Índios | 19·62 | high |
| 2024-07-30 | 31 | 28 | Male | Palmeira Dos Índios | 19·49 | high |
| 2024-07-30 | 31 | 28 | Male | Palmeira Dos Índios | 34·72 | low |
| 2024-07-30 | 31 | 35 | Male | Palmeira Dos Índios | 21·45 | high |
| 2024-07-30 | 31 | 29 | Female | Igaci | 19·49 | high |
| 2024-07-31 | 31 | 14 | Male | Palmeira Dos Índios | 24·75 | high |
| 2024-08-01 | 31 | 56 | Female | Palmeira Dos Índios | 27·78 | moderate |
| 2024-08-01 | 31 | 57 | Female | Palmeira Dos Índios | 21·75 | high |
| 2024-08-01 | 31 | 38 | Male | Palmeira Dos Índios | 34·89 | low |
| 2024-08-01 | 31 | 10 | Male | Palmeira Dos Índios | 21·53 | high |
| 2024-08-02 | 31 | 38 | Female | Palmeira Dos Índios | 18·74 | high |
| 2024-08-02 | 31 | 28 | Female | Palmeira Dos Índios | 35·65 | low |
| 2024-08-02 | 31 | 16 | Female | Palmeira Dos Índios | 18·76 | high |
| 2024-08-03 | 31 | 25 | Female | Palmeira Dos Índios | 28·15 | moderate |
| 2024-08-04 | 32 | 24 | Female | Palmeira Dos Índios | 19·45 | high |
| 2024-08-05 | 32 | 16 | Female | Palmeira Dos Índios | 36·88 | low |
| 2024-08-06 | 32 | 51 | Male | Palmeira Dos Índios | 21·02 | high |
| 2024-08-06 | 32 | 28 | Male | Palmeira Dos Índios | 34·94 | low |
| 2024-08-08 | 32 | 28 | Female | Palmeira Dos Índios | 18·68 | high |
| 2024-08-11 | 33 | 28 | Male | Palmeira Dos Índios | 20·21 | high |
| 2024-08-11 | 33 | 62 | Female | Palmeira Dos Índios | 16·55 | high |
| 2024-08-11 | 33 | 32 | Male | Palmeira Dos Índios | 36·45 | low |
| 2024-08-11 | 33 | 47 | Female | Palmeira Dos Índios | 26·87 | moderate |
| 2024-08-12 | 33 | 44 | Female | Palmeira Dos Índios | 18·84 | high |
| 2024-08-12 | 33 | 77 | Male | Palmeira Dos Índios | 19·93 | high |
| 2024-08-13 | 33 | 49 | Male | Palmeira Dos Índios | 32·81 | low |
| 2024-08-13 | 33 | 55 | Female | Palmeira Dos Índios | 18·8 | high |
| 2024-08-13 | 33 | 76 | Male | Palmeira Dos Índios | 33·26 | low |
| 2024-08-13 | 33 | 44 | Male | Palmeira Dos Índios | 36·78 | low |
| 2024-08-13 | 33 | 58 | Female | União Dos Palmares | 36·43 | low |
| 2024-08-13 | 33 | 30 | Female | Maceió | 32·05 | low |
| 2024-08-13 | 33 | 18 | Female | Palmeira Dos Índios | 23·55 | high |
| 2024-08-15 | 33 | 54 | Male | Palmeira Dos Índios | 30·41 | low |
| 2024-08-15 | 33 | 21 | Male | Maceió | 34·79 | low |
| 2024-08-15 | 33 | 58 | Male | Palmeira Dos Índios | 16·37 | high |
| 2024-08-15 | 33 | 29 | Female | Palmeira Dos Índios | 19·21 | high |
| 2024-08-15 | 33 | 31 | Female | Palmeira Dos Índios | 34·71 | low |
| 2024-08-15 | 33 | 20 | Female | Palmeira Dos Índios | 35·34 | low |
| 2024-08-15 | 33 | 23 | Female | Palmeira Dos Índios | 24·45 | high |

* EW - Epidemiological Week

** Ct_value - cycle threshold value

# Considering the viral load, Ct values <25, 25–30, and >30 were categorized as high, moderate, and low, respectively, according to Mishra and collaborators^1^.

**References**

1 Mishra B, Ranjan J, Purushotham P, Kar P, Payal P, Saha S, et al. Comparison of Cycle Threshold and Clinical Status Among Different Age Groups of COVID-19 Cases. *Cureus*. 2022;**14**:e24194. https://doi.org/10.7759/cureus.24194
