# Supplementary material for: Arrival of Oropouche Virus in a Nonendemic Area in Northeastern Brazil, 2024
Source: J Med Virol. 2025 Dec 29;98(1):e70780. doi: 10.1002/jmv.70780 (PMC12746540; doi:10.1002/jmv.70780)
Supplement: Supplementary file 4 — Table S3: Cell culture supernatant (1st Passage) processed by RT‐qPCR and Indirect Immunofluorescence. [file JMV-98-e70780-s001.docx]

**Table S3 - Cell culture supernatant (1st Passage) processed by RT-qPCR and Indirect Immunofluorescence.**

| **LACEN identification** | **DPI*** | **RT-qPCR result** | **Cycle Threshold** | **IFA**** | **LAPEVI strain** |
| --- | --- | --- | --- | --- | --- |
| Sample 1 | 3 | Positive | 37.95 | Positive | OROV AL01 |
| Sample 2 | 3 | Positive | 14.30 | Positive | OROV AL02 |
| Sample 3 | 3 | Positive | 13.28 | Positive | OROV AL03 |
| Sample 4 | 3 | Positive | 13.12 | Positive | OROV AL04 |
| Sample 5 | 3 | Positive | 13.91 | Positive | OROV AL05 |
| Sample 6 | 3 | Positive | 13.43 | Positive | OROV AL07 |
| Sample 7 | 3 | Positive | 12.72 | Positive | OROV AL11 |
| Sample 8 | 3 | Positive | 13.18 | Positive | OROV AL12 |
| Sample 9 | 3 | Positive | 11.26 | Positive | OROV AL13 |
| Sample 10 | 3 | Positive | 12.47 | Positive | OROV AL14 |
| Sample 11 | 3 | Positive | 12.83 | Positive | OROV AL15 |
| Sample 12 | 3 | Positive | 11.87 | Positive | OROV AL16 |
| Sample 13 | 3 | Positive | 11.87 | Positive | OROV AL17 |
| Sample 14 | 3 | Positive | 14.12 | Positive | OROV AL18 |

* DPI - days post infection

** IFA - Indirect immunofluorescence assay
